# Usefulness of ^11^C-methionine positron emission tomography for detecting intracranial ameloblastic carcinoma: A case report

**DOI:** 10.3892/ol.2014.2352

**Published:** 2014-07-15

**Authors:** AKIRA TEMPAKU, YOSHINOBU TAKAHASHI, HIDETOSHI IKEDA, SHIGERU YAMAUCHI, TAKASHI GOTOH, NOBUYUKI BANDOH, SYUJIROU MAKINO, TAKUJI SHIMADA, HAJIME KAMADA

**Affiliations:** 1 Department of Neurosurgery, Hokuto Hospital, Hokkaido 080-0833, Japan; 2 Department of Otolaryngology, Hokuto Hospital, Hokkaido 080-0833, Japan; 3 Department of Oral and Maxillofacial Surgery, Hokuto Hospital, Hokkaido 080-0833, Japan; 4 Department of Plastic Surgery, Hokuto Hospital, Hokkaido 080-0833, Japan

**Keywords:** ameloblastic carcinoma, intracranial extension, methionine-labeled positron emission tomography

## Abstract

Ameloblastic carcinoma, secondary type, is an extremely rare odontogenic malignant tumor. The present study reports the case of a 58-year-old male with ameloblastic carcinoma that extended into the intracranial space close to the internal carotid artery. Surgical excision was performed, as headaches were being caused via compression by the mass. Small remnants of the tumor remained surrounding the internal carotid artery following surgical resection. Although the remnant tissue was not detected on magnetic resonance imaging or 18F-fluorodeoxyglucose (FDG)-positron emission tomography (PET), it was clearly visualized on ^11^C-methionine PET in the early post-operative follow-up period. No neurological deficits were exhibited during the follow-up period, and ^11^C-methionine PET was able to detect the remnant lesion distribution in the intracranial space. The current study presents a rare case of ameloblastic carcinoma that extended into the intracranial space. In addition, several diagnostic imaging tools were compared in order to determine the most suitable imaging modality. At present, the patient is continuing a therapeutic course of radiation and evident mass reduction has been observed. However, the therapeutic effects are currently under consideration. To the best of our knowledge, this is the first study on the effectiveness of using ^11^C-methionine PET for detecting ameloblastic carcinoma with intracranial extension.

## Introduction

Ameloblastic carcinoma, peripheral secondary type, is a rare odontogenic carcinoma (1), and few cases of the intracranial extension of these tumors have been reported. Ameloblastic carcinoma is defined as a rare malignant odontogenic tumor that retains the histological features of ameloblastoma and also exhibits cytological features of malignancy (1–5). ^11^C-methionine positron emission tomography (PET) is the most common method used to detect intracranial malignancies with high resolution, as ^11^C-methionine is preferentially absorbed in tissue with highly active amino acid and protein synthesis. Protein synthesis is initiated at the first methionine amino acid and the level of ^11^C-methionine uptake indicates the level of protein synthesis. As the normal brain tissue has low levels of protein synthesis activitiy, intracranial malignant lesions usually uptake much higher levels of ^11^C-methionine. Predominantly, ^11^C-methionine PET is applied to detect malignant lesions in the head and neck region and has never been used for the detection of ameloblastic carcinoma, due to the rarity of the tumor (6–8). The current study presents a rare case of maxillary ameloblastic carcinoma, peripheral secondary type, extending into the right middle cranial fossa. In addition, the value of ^11^C-methionine PET is discussed with regard to detecting ameloblastic carcinoma. The patient provided written informed consent.

## Case report

A 58-year-old male was admitted to the Hokuto Hospital (Hokkaido, Japan) with a moderate headache and dizziness. The patient had a normal level of consciousness. No evident central nervous system disorders were observed. The patient had a history of ameloblastoma, which had been treated 12 years prior to admission. The post-operative follow-up for this occurrence had been discontinued by the Obihiro-Kosei General Hospital (Obihiro, Japan).

**Figure 1:**
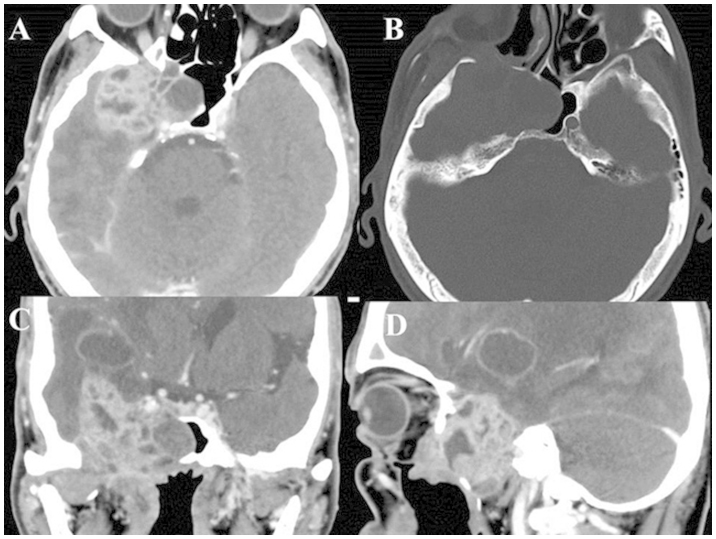
CT scan obtained on admission. (A) Axial, (C) coronal and (D) sagittal enhanced CT scans showing a mass occupying the maxillary sinus extending to the temporal base. (B) Bone imaging showing a right middle cranial base deficiency. CT, computed tomography.

**Figure 2:**
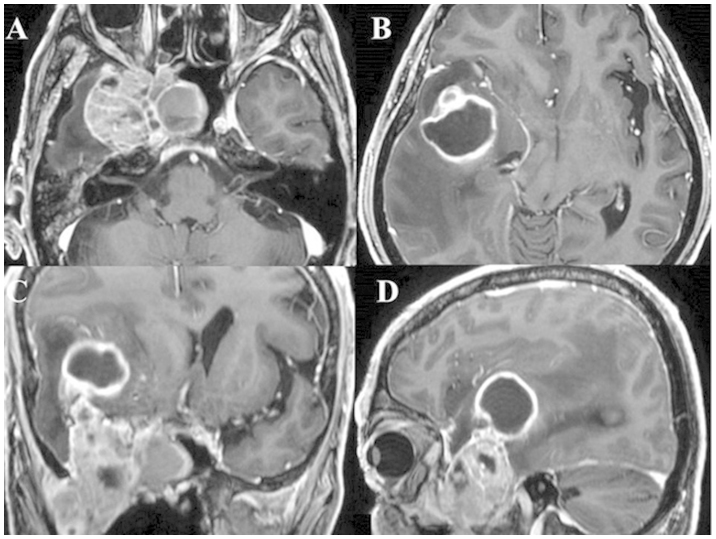
Enhanced magnetic resonance imaging performed on admission. (A and B) Axial, (C) coronal and (D) sagittal views showing a solid mass extending from the maxillary sinus to the middle cranial base.

**Figure 3:**
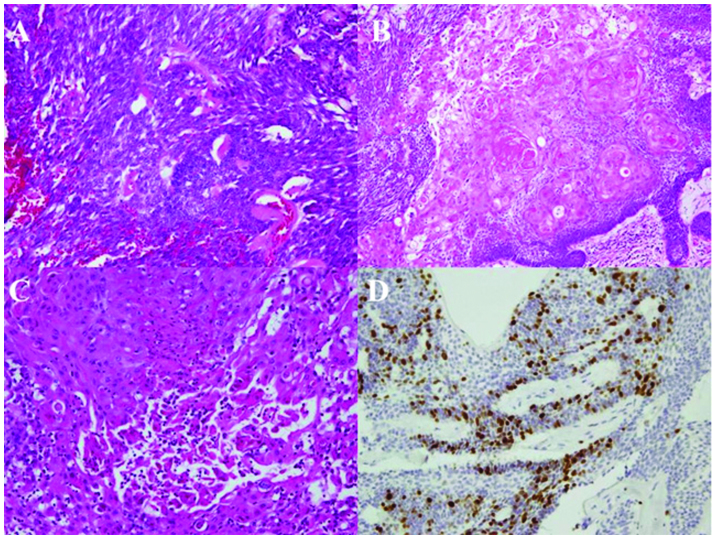
Immunohistochemical study. Hematoxylin and eosin staining results showing (A) a level of high cellularity (magnification, ×200), with (B) hyperkeratosis (magnification, ×100) and (C) necrosis (magnification, ×200). (D) MIB-1 staining (magnification, ×200). The proportion of MIB-1-positive cells was ~25%.

**Figure 4:**
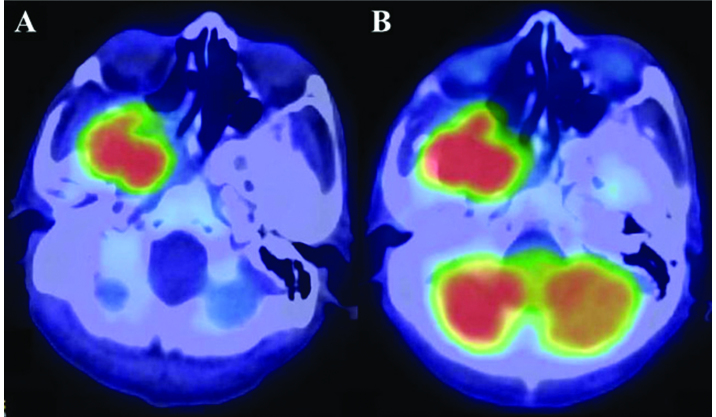
Positron emission tomography scan prior to surgery showing a high accumulation of (A) ^11^C-methionine and (B) 5-fluorodeoxyglucose in the mass lesion.

Computed tomography (CT) and magnetic resonance imaging (MRI) revealed a mass lesion in the right infratemporal fossa, middle cranial space and upper maxilla, with a wide-range bone defect in the right middle skull base (Fig. 1 and 2). A histopathological specimen was obtained from the tumor in the oral cavity. Hematoxylin and eosin (HE) staining demonstrated marked atypia, high cellularity, hyperkeratosis and necrosis surrounded by the typical ameloblastoma histology (Fig. 3). MIB-1 staining showed 25% positive cells in the lesion (Fig. 3). These findings indicated a diagnosis of ameloblastic carcinoma. For contrast agents, 185 MBq 18F-fluorodeoxyglucose and 370 MBq ^11^C-methionine (10 mCi) were injected intravenously as bolus injections. PET revealed a high level of accumulation of 18F-fluorodeoxyglucose (FDG) and ^11^C-methionine in the mass (Fig. 4). No evident lesions were detected, except for the brain mass, on enhanced whole-body MRI or PET.

**Figure 5:**
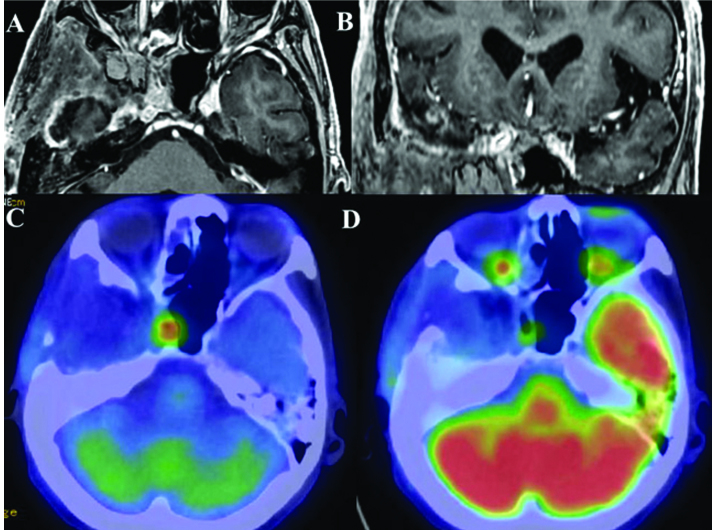
Post-operative examination. (A, B) Enhanced MRI and (D) FDG-PET were unable to visualize the remnant tissue. (C) ^11^C-methionine-PET clearly revealed the remnant tissue of the ameloblastic carcinoma around the right internal carotid artery.

A surgical resection of the tumor was performed. Almost all the tumor components were removed, except for a small amount of remnant tissue around the right internal carotid artery. During the early post-operative follow-up period, although neither FDG-PET nor enhanced MRI could visualize the residual tissue, ^11^C-methionine PET was able to detect the tumor remnant (Fig. 5). At present, the patient is continuing a therapeutic course of radiation and evident mass reduction has been observed. However, the therapeutic effects are currently under consideration.

## Discussion

According to the 2005 histological classification by the World Health Organization (WHO), ameloblastic carcinoma is classified into the primary type, the intraosseous secondary type and the peripheral secondary type (1,2). Whereas primary-type ameloblastic carcinoma develops *de novo*, secondary-type ameloblastic carcinoma is derived from the malignant transformation of ameloblastoma due to repeated inflammatory stimulation (3). Ameloblastic carcinoma exhibits cytological atypia, with or without metastasis (4,5). In the present study, due to the patient’s past history of ameloblastoma and the findings of histopathological malignancy, a diagnosis of ameloblastic carcinoma, peripheral secondary type, was made. Furthermore, the ameloblastic carcinoma had extended into the intracranial space. Although odontogenic tumors typically arise from oral cavity tissues, one study in the English literature has indicated that these lesions can grow in the head and neck region (9). Furthermore, a small number of studies have also reported the intracranial extension of ameloblastoma (10–14), while a few others have presented cases of ameloblastic carcinoma with extension into the intracranial space or cranial bone (15–17).

Enhanced CT or MRI and/or FDG-PET are generally used to detect the regrowth or metastasis of tumors. In contrast, X-ray, plain CT and enhanced CT are usually applied in cases of malignant ameloblastoma or ameloblastic carcinoma. To the best of our knowledge, the extension of these tumors into the intracranial space is generally detected using plain or enhanced CT (10–14,16). MRI is also useful for detecting the intracranial extension of ameloblastic carcinoma (16). Furthermore, FDG-PET is often applied to assess systemic metastasis (17,18) and skull bone metastasis (17) of ameloblastic carcinoma. However, no previous studies have reported the value of methionine PET for treating ameloblastic carcinoma.

Generally, intracranial malignant lesions are identified on enhanced CT or MRI. Although whole-body FDG-PET is useful for detecting the systemic distribution of malignant lesions (19–22), it is difficult to evaluate small intracranial lesions by this method, as normal brain tissues also exhibit the uptake of FDG to a certain extent (23,24). As protein synthesis is upregulated in tumor cells compared with that observed in the central nervous system, methionine PET is useful for visualizing the clear border of intracranial malignant lesions in cases of glioma (25). In addition, a positive association has been reported between the efficiency of methionine accumulation and the MIB-1 index, an indicator of the proliferation of malignancy, in the setting of rectal cancer metastasis (26). In previous studies, FDG-PET has been described as a diagnostic tool for detecting ameloblastic carcinoma (17–19). The present study confirmed that methionine PET is superior to FDG-PET in detecting intracranial ameloblastic carcinoma, with remarkable sensitivity. In the present case, only methionine PET was able to detect the extremely small amount of remnant tissue of the post-operative ameloblastic carcinoma surrounding the internal carotid artery (Fig. 5C and D). To the best of our knowledge, there have been no previous studies regarding the effectiveness of methionine PET in diagnosing ameloblastic carcinoma. In this case, the accumulation of methionine in the ameloblastic carcinoma lesion was clearly observed on a PET scan (Fig. 4A and 5C). These findings indicate that methionine PET is a more useful diagnostic tool than FDG-PET for detecting intracranial ameloblastic carcinoma.
